# Synthesis and Ultrafast Broadband Optical Limiting Properties of a Two-Branched Twistacene

**DOI:** 10.3390/molecules27113564

**Published:** 2022-06-01

**Authors:** Yanbing Han, Jinchong Xiao, Xingzhi Wu, Yuxiao Wang, Xueru Zhang, Yinglin Song

**Affiliations:** 1 Department of Physics, Harbin Institute of Technology, Harbin 150001, China; hanyanbing1990@126.com (Y.H.); wangyx@hit.edu.cn (Y.W.); xrzhang@hit.edu.cn (X.Z.); 2 Key Laboratory of Chemical Biology of Hebei Province, College of Chemistry and Environmental Science, Hebei University, Baoding 071002, China; jcxiaoicas@163.com; 3 Jiangsu Key Laboratory of Micro and Nano Heat Fluid Flow Technology and Energy Application, School of Physical Science and Technology, Suzhou University of Science and Technology, Suzhou 215009, China; wuxingzhi@usts.edu.cn

**Keywords:** twistacenes, optical limiting, two photon absorption, excited singlet state absorption

## Abstract

A novel two-branched twistacene (**PyDN**) has been designed and synthesized for application on ultrafast optical limiting. This twistacene exhibits excellent two photon absorption and two photon absorption-induced excited singlet state absorption, which was systematically investigated with a femtosecond Z-scan experiment, transient absorption spectrum, and two-photon excited fluorescence experiments. The admirable two photon absorption is attributed to the high degree of π electron delocalization in twistacene which is caused by introduction of two strong donors. The excited singlet state absorption cooperates with two-photon absorption to provide an excellent ultrafast optical limiting behavior with high linear transmittance, where the thresholds are 2.3–5.3 mJ/cm^2^ in the spectral region of 532–800 nm of femtosecond laser and 133 mJ/cm^2^ for picosecond pulse at 532 nm. These thresholds are lower than that of most of the optical limiters reported previously, which indicates **PyDN** is a promising candidate for ultrafast optical limiting.

## 1. Introduction

With the advancement of advanced laser techniques, ultrafast lasers have been widely applied to various fields such as machining, energy conversion, imaging, communications, surgery, and medicine [1,2,3]. Developing optical limiting (OL) materials to protect human eyes and sensitive instruments from high-intensity ultrafast lasers damage is increasingly important. In recent years, great efforts have been devoted to various OL materials such as graphene families [4,5], nanomaterials [6,7], organic compounds [8,9], and so on [10]. Among them, organic π-conjugated compounds have received more attention due to their structural flexibility, the easy fabrication of devices and strong nonlinear optical absorption. For most OL materials, optical nonlinear response is based on nonlinear scattering or excited triplet state absorption of materials [11,12]. These materials are usually highly efficient for long laser pulses (nanoseconds), but are weak for ultrashort laser pulses (femtoseconds and picoseconds for examples). In this case, utilizing two-photon absorption (TPA) induced excited singlet state absorption to realize ultrafast broadband OL behavior is one feasible way to avoiding this disadvantage. Moreover, if the lifetime of the excited-state lifetime in these materials falls into the long pulse duration of the laser, an OL response for different laser pulses could be realized. The TPA is a broadband absorption behavior and can cover a wide spectral region from visible to near-infrared. Together with a broadband excited state absorption (ESA), OL performance will be significantly improved under the various wavelengths, which is a key factor to realize the broadband response to different input laser wavelengths. In addition, the zero contribution of one photon absorption in this process will provide a high optical transparency, avoiding the loss of light under low laser beam intensity caused by linear absorption [13,14,15,16]; however. constructing these materials remains a challenge. 

Twistacenes, as a member of polyclic aromatic hydrocarbons (PAHs) families, contain rigid pyrene units and terminal linked acenes [17,18], which possess a very large π conjugate system and exhibit excellent electro-optical activity [19,20,21,22]. Interestingly, the formed twistacenes exhibit the excellent nonlinear absorption (NLA) properties, which are favorable for OL application, and have attracted our attention. Chen et al. found that end-modified unsymmetrical twistacenes possessed broadband excited state absorption behavior [23]. Then, Wu et al. reported a series of spindle-type conjugated twistacenes dimer, where the TPA-induced excited singlet state absorption of these twistacenes was investigated and confirmed. Moreover, extending the π-conjugated framework in these twistacenes can enlarge the ESA on singlet state, while it retains its broad excited-state absorption spectrum [24]. Song et al. reported a class of phenylacetylene-functionalized twistacenes and twistfuranacenes, and found that the two-photon absorption performance of materials was heavily dependent on the terminal substituted group. The triphenylamine decorated twistacenes exhibited enhanced two-photon absorption behavior, which was ascribed to the introduction of a strong donor into the twistacene unit, which efficiently improved the degree of delocalization of π electrons in twistacenes [25]. These observations inspired us to construct new twistacenes with strong a NLA ability for ultrafast OL application.

**Scheme 1:**
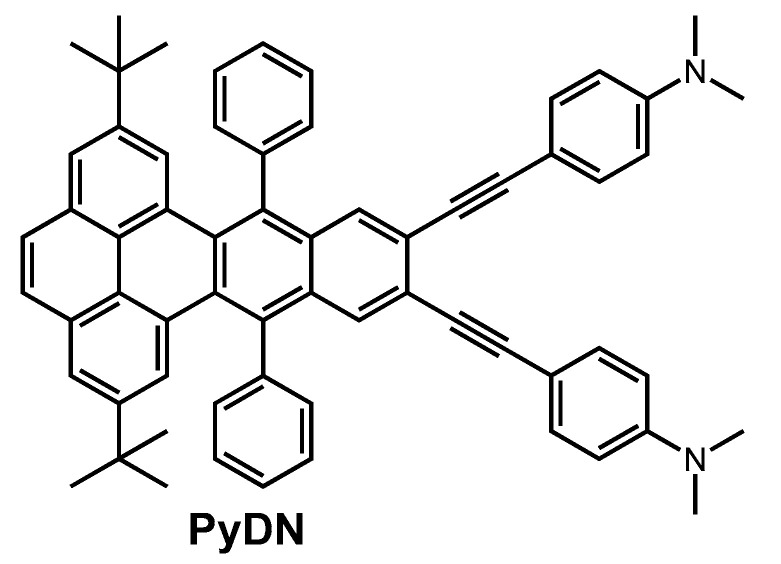
Molecular structures of **PyDN**.

In this work, we designed and synthesized a novel twistacene (**PyDN** in Scheme 1), which contains of a twistacene unit and two strong donors (*N*,*N*-dimethylaniline), and systematically investigated the TPA and excited-state dynamics of **PyDN** with a femtosecond Z-scan experiment, transient absorption, and two-photon excited fluorescence experiments. Benefiting from the good overlap of TPA and ESA, **PyDN** exhibits excellent ultrafast broadband OL performance in femtoseconds and picoseconds regimes.

## 2. Results and Discussion

### 2.1. Molecular Design and Synthesis

**Scheme 2:**
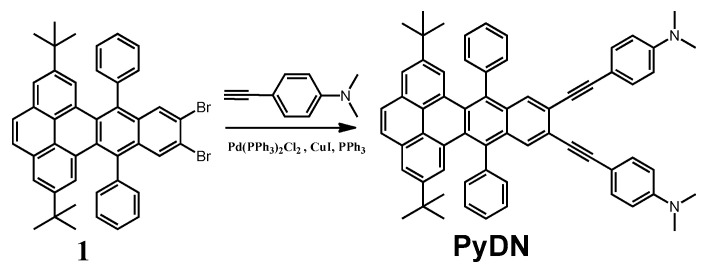
Synthetic route to **PyDN**.

Nonlinear optical properties of organic materials originate from the efficient delocalization of the π-electron. Extending π-conjugated frameworks of a system and introduction of donor or acceptor groups are well-known strategies to improve the NLA property of materials [16,26]. Previous reports showed that the introduction of a strong donor into the twistacene unit could efficiently enhance two-photon absorption capacity in one-branched twistacenes [25]. In addition, a multi-branched structure could provide a larger conjugate system than a one-branched structure, which is expected to improve the OL performance of materials [27,28]. Hence, we designed a two-branched twistacene structure by decorating the strong donor group (*N,N*-dimethylaniline) with acetylene bond as π bridge in adjacent position. This structure possesses an extended π-conjugated system and strong donor, which is expected to improve the OL performance of twistacene. A synthetic route to **PyDN** is depicted in Scheme 2. **PyDN** was prepared from disubstituted twistacene **1** and 4-ethynyl-*N,N*-dimethylaniline, catalyzed by Pd(PPh_3_)_2_Cl_2_ via a Sonogashira reaction. **PyDN** was characterized by ^1^H NMR, ^13^C NMR spectroscopy and mass spectroscopy (See the Supplementary Material).

### 2.2. Electronic Structure Analysis

**Figure 1:**
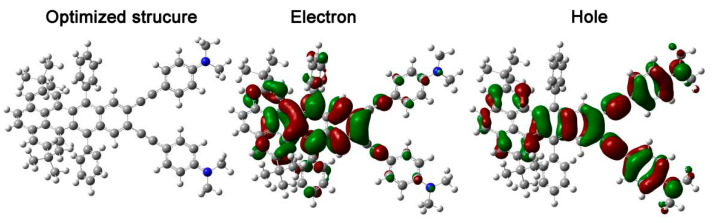
Optimized structure and the natural transition orbitals in the first excited state (S1) of **PyDN**.

To gain insight into the NLA properties of **PyDN**, theoretical calculations were conducted using a Gaussian 09 software package [29]. The ground states molecule were optimized using the B3LYP/6-31G(d,p) level of density functional theory (DFT), and the excitation energy was calculated by using the time-dependent density functional theory (TD-DFT) at same level. After that, the natural transition orbitals (NTOs) calculation was performed to describe the electronic structure characteristics on the excitation states. The calculated result is illustrated in Figure 1. 

The optimized structures show these two modified groups are almost coplanar with the terminal acene structure of twistacene unit. Even though these two modified groups are decorated in adjacent positions of one benzene ring of a twistacene, the introduction of acetylene bonds as a π bridge results in almost no steric hindrance effect between both modified groups. This planar conformation does not cause the twisted intramolecular charge transfer, which is beneficial to the enhancement of TPA [30]. The result of NTOs shows that hole orbital is mainly localized on whole molecular skeleton including the twistacene unit and two modified groups, whereas the distribution of electron orbitals is more localized on the twistacene unit. These observations show that the absorption mainly originates from π–π* transitions, including a certain amount of π electron transfer from modified group to twistacene unit in the process of absorption. The distribution of hole orbitals indicates that the whole molecular skeleton participates in the NLA process, thus resulting in larger delocalization area, which is conducive to the enhancement of TPA. Moreover, transferring a certain amount of π electrons to a twistacene will activate the activity of twistacene with a large conjugated system, which is a benefit to π electron delocalization. Compared withs other twistacenes [23,25], the two-branched twistacene **PyDN** possess a higher degree of π electron delocalization, which is mainly caused by the extending conjugated plane of the system. To further research the substituent effects, a class of similar twistacenes are designed and calculated for comparison, and the calculated results are illustrated in Figure S2. The distributions of orbitals in these three twistacenes are wider than their corresponding one-branched structures [25], indicating the two-branched architectures can provide more of an NLA response. An obvious charge separation in NTOs can be observed in the twistacene with strong acceptor moieties (4-nitrobenzene), which will provide a small distribution of delocalized π electrons and is unfavorable to the enhancement of NLA [30]. In addition, the charge transfer from substituent group to twistacene unit is enhanced by increasing the electron-donating ability of substituent groups, which will be better to activate the large conjugated twistacene units and cause a higher degree of π electrons delocalization. These results show that **PyDN** possesses a high degree of π electron delocalization, and thus, it could maybe provide excellent NLA [16,26]. 

### 2.3. UV-Vis Absorption and Emission

**Figure 2:**
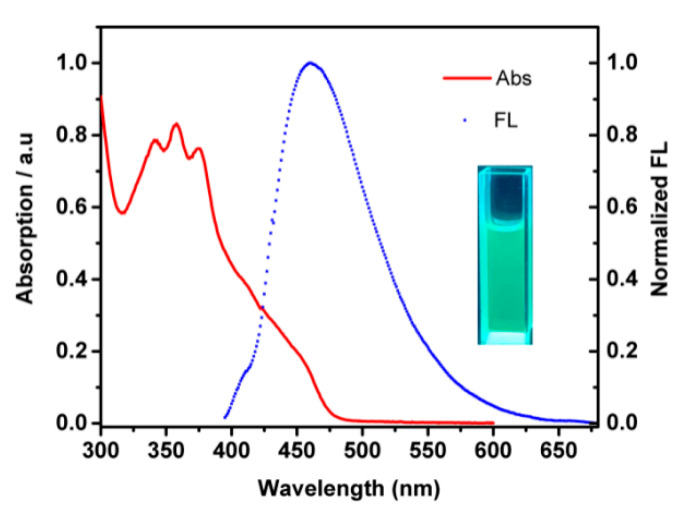
UV-vis absorption (red line) and emission (blue dots) spectra of **PyDN** in toluene. Insets is photograph achieved under black back light with excitation at 365 nm in toluene.

The UV-vis absorption and emission spectra of **PyDN** were investigated in toluene solution (1×10^−5^ M) at ambient condition. As shown in Figure 2, compounds of **PyDN** show obvious absorption in the spectral region of 300–481 nm with absorption bands at 342 nm, 359 nm, and 375 nm, which are assigned to the n–π* and π–π* transition of the skeleton. Compared with single modify group decorated twistacenes [25], there is a slight red shift, which is caused by the extending conjugated system. When excited at 359 nm, **PyDN** emitted light centered at 459 nm with quantum yields (ΦF) of 0.38, using 9,10-diphenylanthracene (ΦF = 0.95 in ethanol) as a standard. The optical energy gaps (Eg) were calculated to be 2.60 eV, according to the absorption edges at 480 nm in toluene.

### 2.4. Open Aperture Z-scan and Transient Absorption Spectrum Experiments

**Figure 3:**
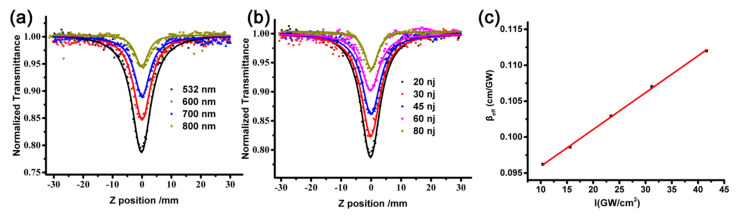
Femtosecond open aperture Z-scan data of **PyDN** under different wavelengths (**a**) and different incident laser energies at 532 nm (**b**); (**c**) incidental laser intensity dependence of efficient NLA coefficient. The different colored dots are the experimental data and solid lines represent the theoretical fitting.

NLA response of **PyDN** in toluene with a concentration of 2.4×10^−3^ M was investigated by an open aperture Z-scan experiment. Yb:KGW fiber laser equipment (Light Conversion, PHAROS-SP) with an optical parametric amplifier (OPA, Light Conversion), which delivers tunable wavelength with ~190 fs pulses, was used as a laser light source, where laser frequency was set at 20 Hz to exclude NLO response from thermal effect. The background signal of toluene solvent was tested to guarantee an NLA signal in the Z-scan that comes from **PyDN**. A broadband NLA is more favorable for the application of ultrafast OL, hence NLA capacity of **PyDN** was investigated in a wide optical window from 532 nm to 800 nm. The transmittance of the sample was recorded by a power meter, and the experimental data were shown in Figure 3a. **PyDN** possesses excellent reverse saturable absorption behavior in the wide spectral region, suggesting that **PyDN** is a good broadband optical limiting material. To investigate the mechanism of absorption, the open-aperture Z-scan experiments with different input intensities of laser beams were carried out at 532 nm and shown in Figure 3b. 

The nonlinear absorption coefficient at can be calculated by using the following equation [31],

(1) $$T(z)=\sum_{m=0}^{\infty }\frac{{\left[-\beta_{eff}IL_{eff}/(1+z^{2}/z_{0}^{2})\right]}^{m}}{{\left(m+1\right)}^{3/2}}$$

where *I* is the input intensity of the laser beam, *β_eff_* is effective NLA coefficients, *α*_0_ is the linear absorption coefficient, $L_{eff}=\left[1-\exp(-\alpha_{0}L)\right]/\alpha_{0}$ represents the sample thickness, and *z_0_* and *z* represent the diffraction length of the beam and the propagation axis coordinate, respectively. The calculated *β_eff_* is nearly proportional, thus increasing the function of input intensity *I* (Figure 3c), which indicates that there is not only mechanism of third-order nonlinear absorption but also a contribution from a higher-order nonlinear response. Considering the zero contribution from one-photon absorption under selected wavelengths, the absorption process of **PyDN** may be assigned to a two-photon (TPA)-induced excited state absorption [14,32]. As three photons are absorbed in the process, TPA-induced excited-state absorption is therefore an effective fifth-order nonlinear absorption.

**Figure 4:**
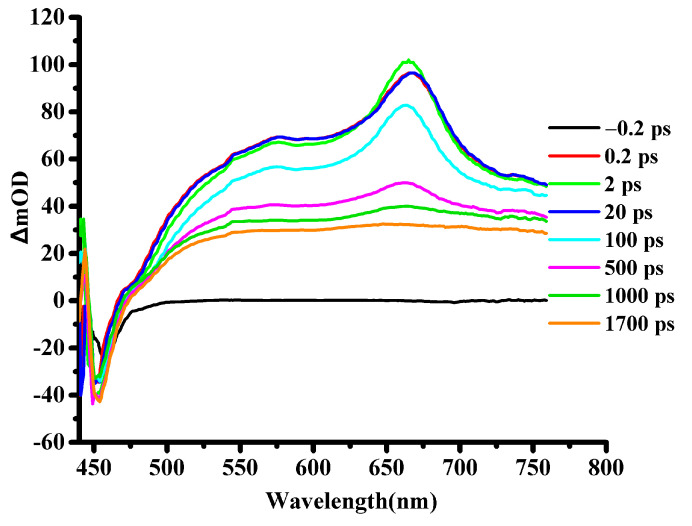
Femtosecond transient absorption spectrum.

To validate this absorption process, a femtosecond transient absorption experiment was carried out to investigate the dynamics of excited state in **PyDN**, and the wavelength of pump light was set to 400 nm to ensure that the sample can be efficiently excited. Moreover, the probe beam is the white light produced in a 2 mm thick Ti sapphire plate. As shown in Figure 4, **PyDN** exhibits a broadband excited state absorption (ESA) behavior (OD > 0) in a spectral region from 476 nm to 760 nm with a strong absorption peak at 662 nm. The negative signal centered at 458 nm is ascribed to the superposition of stimulated emission signal with ESA (Figure 2). During the entire delay time, the curve shape of ESA remains the same with a time depend decay of OD, indicating that the ESA originates from an excited singlet state and there is no additional transition process In addition, the decay of extracted dynamics traces at 458 nm is similar to that at 662 nm (Figure S1), suggesting that the ESA is derived from the fluorescent states and there is no additional transition process occurring [33,34,35]. This ESA behavior is appreciated in ultrafast OL.

The dynamics process can be well fitted throughout the global analysis and two processes were successfully extracted, with a fast process with a time constant of 0.38 ps and a slow process with a lifetime of 3.6 ns (selected dynamic traces and fitting process in Figure S1). Under photoexcitation, the whole transition process can be described as **PyDN** first being pumped to an excited state (S1) by absorbing a photon, then other photons are absorbed to transfer to a higher excited state instantaneously (ESA process). After that, molecules in a higher excited state decay to S1 through the vibrational relaxation of the hot exciton with a lifetime of 0.38 ps, and finally back to the ground state with a lifetime of 3.6 ns. To further validate the process, a transient fluorescence experiment of **PyDN** in toluene was performed. The dynamic curve of fluorescence can be well-fitted by using a mono-exponential function with a lifetime of 3.5 ns (Figure S3), which is in good agreement with the long lifetime in TAS. This result provides more proof to excited singlet state absorption. 

These results imply that the NLA of **PyDN** originates from TPA and TPA-induced ESA in a spectral region from 532 nm to 800 nm. Based on this process, the absorption coefficient can be presented with following equation [36],

(2) $$\alpha =\alpha_{0}+\beta I+\gamma I^{2}$$

where *β* and *γ* represent the TPA coefficient and three-photon absorption coefficient (TPA-ESA absorption), respectively. Additionally, the TPA cross-sections can be calculated by using the equation: σ_TPA_ = *hωβ*/N, where *hω* represents the photon energy at the selected wavelength and N is the number density of **PyDN** in toluene. The TPA cross-sections and three-photon absorption coefficient at the selected wavelengths can be obtained by fitting the open-aperture Z-scan data using Equations (1) and (2) again. As shown in Table 1, **DyPN** exhibits good two-photon absorption behavior with a maximum absorption cross-section of 2476 GM. 

### 2.5. Two-Photon-Excited Fluorescence (TPEF) Experiment

**Figure 5:**
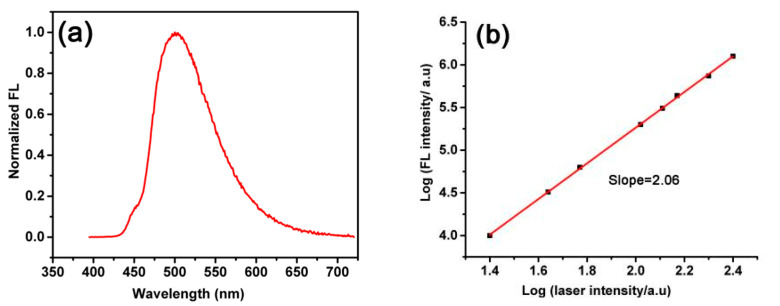
(**a**) Normalized two-photon-excited emission spectra in toluene; (**b**) the logarithmic plots of power-squared dependence of two-photon-excited fluorescence intensity on the laser intensity.

To further investigate the TPA behavior of **PyDN**, the two-photon-excited fluorescence (TPEF) experiment was conducted. **PyDN** was freshly dissolved in toluene with a concentration of 1×10^−4^ M for this measurement. The laser source is same with the z-scan experiment, and the repetition rate is set at 6 kHz. The intensity level of the laser beam was carefully controlled to prevent saturation of absorption and photodegradation of **PyDN** in the process of measurement. The excitation wavelength of this measurement was selected in spectral region from 610 nm to 900 nm, as the shorter wavelengths will interfere with the collection of the fluorescence signals, resulting in large discrepancies between the experimental results and real results in our measurement system. **PyDN** exhibits bright and observable emissions by an unfocused femtosecond laser beam at selected wavelengths (Figure 5a). The intensity of emission shows obvious power-squared dependence on the input excitation intensity under selected wavelength, which confirms that the observed fluorescence emission at a selected wavelength is mainly induced by TPA rather than another absorption process (Figure 5b). In addition, the shape and position of fluorescence emission is identical to the one photon excited fluorescence band, which indicates that the final excited states of TPA is the same as that of one photon absorption, and that no excitation process participates in the TPA-induced fluorescence emission.

**Figure 6:**
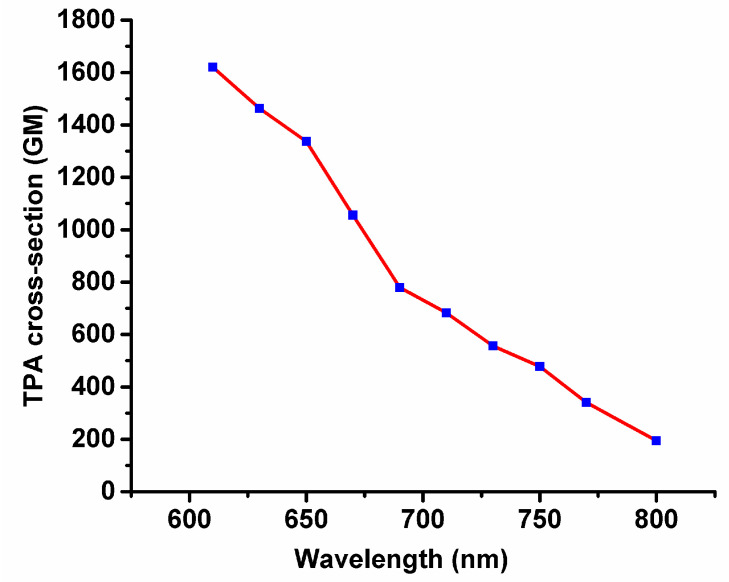
TPA spectra in toluene measured by TPEE method.

The TPA cross-sections of **PyDN** were obtained by using fluorescein in water at pH 11 as a standard, and were calculated using the following equation [37]:

(3) $$\delta_{s}=\frac{S_{s}C_{r}\Phi_{r}\eta_{r}}{S_{r}C_{s}\Phi_{s}\eta_{s}}\cdot \delta_{r}$$

where *s* and *r* represent the measured sample and reference sample, respectively. *S* is the detected fluorescence integral area, *C* is the concentration of the sample, *Φ* represents the fluorescence quantum yield of sample, *η* represents the collection efficiency of the experimental instruments. The **PyDN** sample and the reference sample were placed into a 10 mm spectroscopic quartz cuvette, and every measurement for these two samples was conducted under the same experimental conditions. The obtained TPA spectra (Figure 6) agrees with that of one photon absorption, which confirms that the transition of TPA is the same as that of one photon absorption (S0–S1 transition). The maximum value of the TPA cross-section is 1620 GM at 610 nm, which is larger than the corresponding value from Z-scan experiment at same wavelength. This observation may be resulted from the partial bleaching of high concentration samples used in Z-scan experiment [38]. Compared with single modify group decorated twistacenes [25], the TPA cross-section of **PyDN** from both methods was been greatly improved, which may have been caused by extending the conjugate system introduced by the strong donor decorated two-branched structure. 

### 2.6. Ultrafast Optical Limiting

It is well known that an ideal optical limiting device is expected to possess some characteristics, including high linear transmittance, strong reverse saturated absorption ability, temporally agile response to incident laser pulses, and a broadband response to various wavelengths. Among various NLO mechanisms employed for OL, TPA is a most promising process to meet these requirements, where a zero contribution of linear absorption will make materials transparent to the incident laser pulse under a low intensity, and the quadratic dependence on the input laser intensity can produce good reverse saturated absorption behavior. In addition, an outstanding overlap between the TPA and excited state absorption can efficiently improve the reverse saturated absorption ability of materials to achieve better control to high intensity laser. Under these considerations, **PyDN** possesses the TPA and TPA-induced excited state absorption properties which are excellent candidates for application on ultrafast broadband OLs. 

**Figure 7:**
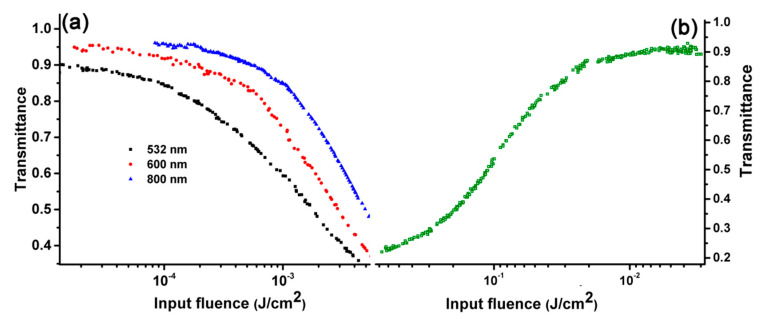
(**a**) Optical limiting behavior of **PyDN** under selected wavelength with a 190 fs pulse; (**b**) optical limiting behavior under 532 nm with a 15 ps pulse.

The OL behavior of an organic solution depends on both the reverse saturated absorption ability of materials and the concentration of organic compounds in a solution. Hence, a nearly saturated **PyDN** solution in toluene was prepared to evaluate the OL performance. The apparatus for OL measurement was similar to the open-aperture Z-scan experiment, where a computer-controlled energy flow modulator consisting of a half wave plate and a linear polarizer was added to modulate the incidental energy flow of the sample. A **PyDN** solution in a 5 mm spectroscopic quartz cuvette was placed in front of lens. Femtosecond laser pulses with wavelengths at 532 nm, 600 nm, and 800 nm were employed to characterize TPA-based optical power that was suppressing properties of **PyDN**. Although the OL performance in femtosecond regime has been mentioned in terms of the open aperture Z-scan experiment, the detailed limiting threshold has not been discussed. The result is illustrated in Figure 7. 

Obviously, high transmittance at low incident energy (T > 90%) quickly decreases with the increasing of incident laser fluence, indicating an effective suppression of a high intensity laser pulse. The limiting threshold (F_th_, defined as the incident fluence where the true transmittance reduces to half of the linear transmittance) is estimated to be 2.3 mJ/cm^2^ (532 nm), 3.2 mJ/cm^2^ (600 nm), and 5.3 mJ/cm^2^ (800 nm). In addition, **PyDN** exhibits an excited singlet state absorption behavior, which breaks the limit of intersystem crossing for application on ultrafast OLs. The optical-power restriction behavior under the irradiation of picosecond laser pulses was also measured and investigated by employing a mode-locked Nd:YAG laser (15 ps) with a repetition rate of 10 Hz. As shown in Figure 7b, **PyDN** also exhibits an excellent OL performance with the picosecond pulse, and the threshold is estimated to be 133 mJ/cm^2^. The ultrafast optical–control performance of PyDN under femtosecond and picosecond laser pulses is comparable, or better than, previously reported graphene oxides [39], semiconductors [40], pyrene-based molecules [41], and hydrazone derivatives [15], indicating that twistacene derivatives are excellent candidates for ultrafast OL applications.

## 3. Materials and Methods

### 3.1. General Method

Materials used in this paper were purchased from commercial companies. Dry triethylamine was obtained by distilling over calcium hydride (CaH_2_). Tetrahydrofuran was distilled over sodium-benzophenone ketyl. ^1^H NMR and ^13^C NMR spectra were recorded in 400 MHz for ^1^H and 100 MHz for ^13^C at room temperature. UV-vis spectra were obtained by using SPECORD 210 plus spectrophotometer. Fluorescence spectra were finished by using HITACHI (F-7000) spectrofluorometer.

### 3.2. Synthesis

A mixture of compound 1 (145 mg, 0.2 mmol), 4-ethynyl-*N*,*N*-dimethylaniline (70 mg, 0.48 mmol, 2.4 equiv), triphenylphosphine (5.6 mg, 0.024 mmol), CuI (4.5 mg, 0.024 mmol) and Pd(PPh_3_)_2_Cl_2_ (14 mg, 0.02 mmol) in a Et_3_N/THF (15 mL/5 mL, *v*/*v* = 1:1) was stirred for 16 h at 80 °C under a nitrogen atmosphere. After cooling to room temperature, brine was added and extracted with dichloromethane. The organic layers were collected, dried with Na_2_SO_4_, and concentrated under reduced pressure. The residue was purified on silica gel column to afford a yellow solid compound **PyDN** (116 mg, 68%). ^1^H NMR (400 MHz, CDCl_3_) δ = 7.99–7.98 (d, *J* = 1.2 Hz, 1H), 7.93–7.87 (s, 7H), 7.70–7.68 (d, *J* = 7.2 Hz, 2H), 7.63–7.56 (m, 4H), 7.53–7.38 (m, 4H), 7.37–7.03 (m, 1H), 6.73–6.71 (d, *J* = 8.8 Hz, 2H), 6.60–6.58 (d, *J* = 8.8 Hz, 2H), 3.05 (s, 6H), 3.00 (s, 6H), 1.16 ((s, 18H)). ^13^C NMR (101 MHz, CDCl_3_) δ = 150.16, 149.57, 147.34, 147.00, 142.31, 141.85, 136.58, 136.01, 135.40, 133.35, 133.03, 132.86, 132.81, 132.62, 130.72, 130.70, 130.21, 129.53, 129.37, 129.31, 129.19, 128.76, 128.40, 127.74, 127.60, 127.42, 126.96, 126.74, 124.17, 123.85, 122.40, 122.17, 120.68, 111.85, 111.31, 110.00, 100.85, 91.97, 87.77, 87.36, 40.25, 34.73, 31.43. MS (MALDI-TOF, *m*/*z*): calculated for C_64_H_56_N_2_, 853.14; Found, 853.17.

## 4. Conclusions

In summary, we have successfully designed and synthesized a two-branched decorated twistacene **PyDN**, and systematically investigated the nonlinear optical properties. The introduction of two strong donors into a twistacene effectively extends the π-conjugated plane and the degree of π electron delocalization, causing excellent nonlinear optical absorption behavior. The absorption mechanism based on TPA and TPA-induced excited singlet state absorption is confirmed by the femtosecond Z-scan experiment, transient absorption, and two-photon excited fluorescence experiments. Benefiting from the spectral overlap between two photon absorption and the excited singlet state absorption, **PyDN** exhibits admirable ultrafast broadband OL performance in femtosecond and picosecond regimes with high transmittance and low thresholds, which is a promising candidate for ultrafast OL applications. In addition, the strategy of constructing twistacene by the introduction of multiple donors is very efficient route to improve the OL performance of materials, and it inspired us to construct new twistacenes for ultrafast OL applications in the future. 

## Figures and Tables

**Table 1:** The parameters of TPA and TPA induced ESA of **PyDN** under selected wavelengths achieved from Z-scan measurements.

	532 nm	600 nm	700 nm	800 nm
β [10^−2^ cmGW^−1^]	9.57	5.00	1.41	0.54
σ_TPA_ [GM]	2476	1147	277	93
γ [10^−4^ cm^3^GW^−2^]	6.50	2.98	2.13	1.21

## Data Availability

The data presented in this study are available in Supplementary Materials.

## Supplementary Materials

The following supporting information can be downloaded at: https://www.mdpi.com/article/10.3390/molecules27113564/s1, Figure S1: Dynamics traces of **PyDN** at 458 nm and 662 nm. The dots represent the experimental data and solid lines were theoretical fitted curves by global analysis; Figure S2: The natural transition orbitals in the first excited state (S1) of designed twistacenes; Figure S3: The emission spectra of PyDN in toluene under different incident laser energies at 400 nm. Insets is that linear dependence of one-photon-excited fluorescence intensity on the laser in-tensity; Figure S4: Fluorescence decay of **PyDN** in toluene. The solid line was fitted lifetime of 3.5 ns; Figure S5: ^1^H NMR spectrum of **PyDN**; Figure S6: ^1^^3^C NMR spectrum of **PyDN**; Figure S7: MALDI-TOF spectrum of **PyDN**.

## Sample Availability

Samples of the **PyDN** are available from the authors.

## References

[1] Mihailov S.J., Hnatovsky C., Abdukerim N., Walker R.B., Lu P., Xu Y., Bao X., Ding H., De Silva M., Coulas D. (2021). Ultrafast Laser Processing of Optical Fibers for Sensing Applications. Sensors.

[2] Lei S., Zhao X., Yu X., Hu A., Vukelic S., Jun M.B.G., Joe H.-E., Yao Y.L., Shin Y.C. (2020). Ultrafast Laser Applications in Manufacturing Processes: A State-of-the-Art Review. J. Manuf. Sci. Eng..

[3] Leburn C.G. (2021). Unifying ultrafast laser imaging and spectroscopy. PhotonicViews.

[4] Lim G.-K., Chen Z.-L., Clark J., Goh R.G.S., Ng W.-H., Tan H.-W., Friend R.H., Ho P.K.H., Chua L.-L. (2011). Giant broadband nonlinear optical absorption response in dispersed graphene single sheets. Nat. Photonics.

[5] Kumar V. (2021). Linear and Nonlinear Optical Properties of Graphene: A Review. J. Electron. Mater..

[6] Xing F., Wang Y., Wang J., Zhou S., Zhao J., Xie Z. (2021). Highly dispersed antimonene oxide quantum dots and their hybrid gel glasses for broadband nonlinear optical limiting. J. Mater. Chem. C.

[7] Sun X., Hu X., Sun J., Xie Z., Zhou S. (2019). Strong optical limiting properties of Ormosil gel glasses doped with silver nano-particles. N. J. Chem..

[8] Liu Z., Sun J., Yan C., Xie Z., Zhang G., Shao X., Zhang D., Zhou S. (2020). Diketopyrrolopyrrole based donor–acceptor π-conjugated copolymers with near-infrared absorption for 532 and 1064 nm nonlinear optical materials. J. Mater. Chem. C.

[9] Sun J., Yuan B., Hou X., Yan C., Sun X., Xie Z., Shao X., Zhou S. (2018). Broadband optical limiting of a novel twisted tetrathiafulvalene incorporated donor–acceptor material and its Ormosil gel glasses. J. Mater. Chem. C.

[10] Dini D., Calvete M.J., Hanack M. (2016). Nonlinear Optical Materials for the Smart Filtering of Optical Radiation. Chem. Rev..

[11] Tutt L.W., Boggess T.F. (1993). A review of optical limiting mechanisms and devices using organics, fullerenes, semiconductors and other materials. Prog. Quantum Electron..

[12] Spangler C.W. (1999). Recent development in the design of organic materials for optical power limiting. J. Mater. Chem..

[13] Lin T.-C., Li M.-L., Liu C.-Y., Tsai M.-Y., Lee Y.-H., Febriani Y., Lin J.-H., Shen Y.-K. (2014). Synthesis and Two-Photon Properties of Multi-Branched Fluorophores Composed of Ladder-Type Conjugated Cores and Functionalized Diquinoxalinylamino Peripheries. Eur. J. Org. Chem..

[14] Xiao Z., Shi Y., Sun R., Ge J., Li Z., Fang Y., Wu X., Yang J., Zhao M., Song Y. (2016). Ultrafast broadband optical limiting in simple pyrene-based molecules with high transmittance from visible to infrared regions. J. Mater. Chem. C.

[15] Jia J., Wu X., Fang Y., Yang J., Guo X., Xu Q., Han Y., Song Y. (2018). Ultrafast Broad-Band Optical Limiting in Simple Hydrazone Derivatives with a Π-Conjugated System: Effect of Two-Photon-Induced Singlet-State Absorption. J. Phys. Chem. C.

[16] Pawlicki M., Collins H.A., Denning R.G., Anderson H.L. (2009). Two-Photon Absorption and the Design of Two-Photon Dyes. Angew. Chem. Int. Ed..

[17] Duong H.M., Bendikov M., Steiger D., Zhang Q., Sonmez G., Yamada J., Wudl F. (2003). Efficient Synthesis of a Novel, Twisted and Stable, Electroluminescent “Twistacene”. Org. Lett..

[18] Jin P., Song T., Xiao J., Zhang Q. (2018). Recent Progress in Using Pyrene-4,5-diketones and Pyrene-4,5,9,10-tetraketones as Building Blocks to Construct Large Acenes and Heteroacenes. Asian J. Org. Chem..

[19] Liu Z., Xiao J., Fu Q., Feng H., Zhang X., Ren T., Wang S., Ma D., Wang X., Chen H. (2013). Synthesis and Physical Properties of the Conjugated Dendrons Bearing Twisted Acenes Used in Solution Processing of Organic Light-Emitting Diodes. ACS Appl. Mater. Interfaces.

[20] Martinez-Abadia M., Antonicelli G., Zuccatti E., Atxabal A., Melle-Franco M., Hueso L.E., Mateo-Alonso A. (2017). Synthesis and Properties of a Twisted and Stable Tetracyano-Substituted Tetrabenzoheptacene. Org. Lett..

[21] Lv B., Xiao J., Zhou J., Zhang X., Duan J., Su W., Zhao J. (2016). Synthesis, Crystal Analyses, Physical Properties, and Electroluminescent Behavior of Unsymmetrical Heterotwistacenes. ACS Appl. Mater. Interfaces.

[22] Wei L., Deng X., Yu X., Li X., Wang W., Zhang C., Xiao J. (2021). Double π-Extended Helicene Derivatives Containing Pentagonal Rings: Synthesis, Crystal Analyses, and Photophysics. J. Org. Chem..

[23] Chen S., Xiao J., Zhang X., Shen X., Liu X., Shen F., Yi Y., Song Y. (2016). Effect of the mismatch structure on crystal packing, physical properties and third-order nonlinearity of unsymmetrical twistacenes. Dyes Pigm..

[24] Wu X., Xiao J., Sun R., Jin T., Yang J., Shi G., Wang Y., Zhang X., Song Y. (2017). Spindle-Type Conjugated Compounds Containing Twistacene Unit: Synthesis and Ultrafast Broadband Reverse Saturable Absorption. Adv. Opt. Mater..

[25] Song T., Han Y., Jin P., Li X., Song Y., Xiao J. (2019). The enhanced two-photon absorption behavior of twistfuranacenes to phenylacetylene-functionalized twistacenes. J. Mater. Chem. C.

[26] He G.S., Tan L.-S., Zheng Q., Prasad P.N. (2008). Multiphoton Absorbing Materials:  Molecular Designs, Characterizations, and Applications. Chem. Rev..

[27] Lin T.-C., Chen Y.-F., Hu C.-L., Hsu C.-S. (2009). Two-photon absorption and optical power limiting properties in femtosecond regime of novel multi-branched chromophores based on tri-substituted olefinic scaffolds. J Mater. Chem..

[28] Ceymann H., Rosspeintner A., Schreck M.H., Mützel C., Stoy A., Vauthey E., Lambert C. (2016). Cooperative enhancement versus additivity of two-photon-absorption cross sections in linear and branched squaraine superchromophores. Phys. Chem. Chem. Phys..

[29] Robb M.A., Cheeseman J.R., Scalmani G., Barone V., Mennucci B., Petersson G.A., Nakatsuji G.A.H., Caricato M., Li X., Hratchian H.P. (2010). Gaussian 09, Revision B.01.

[30] Sun C.-L., Liao Q., Li T., Li J., Jiang J.-Q., Xu Z.-Z., Wang X.-D., Shen R., Bai D.-C., Wang Q. (2015). Rational design of small indolic squaraine dyes with large two-photon absorption cross section. Chem. Sci..

[31] Sheik-Bahae M., Said A.A., Wei T.H., Hagan D.J., Stryland E.W.V. (1990). Sensitive measurement of optical nonlinearities using a single beam. IEEE J. Quantum Electron..

[32] Teran N.B., He G.S., Baev A., Shi Y., Swihart M.T., Prasad P.N., Marks T.J., Reynolds J.R. (2016). Twisted Thiophene-Based Chromophores with Enhanced Intramolecular Charge Transfer for Cooperative Amplification of Third-Order Optical Nonlinearity. J. Am. Chem. Soc..

[33] Kong J., Zhang W., Li G., Huo D., Guo Y., Niu X., Wan Y., Tang B., Xia A. (2020). Excited-State Symmetry-Breaking Charge Separation Dynamics in Multibranched Perylene Diimide Molecules. J. Phys. Chem. Lett..

[34] Sebastian E., Hariharan M. (2021). Null Exciton-Coupled Chromophoric Dimer Exhibits Symmetry-Breaking Charge Separation. J. Am. Chem. Soc..

[35] Hu W., Prasad P.N., Huang W. (2021). Manipulating the Dynamics of Dark Excited States in Organic Materials for Phototheranostics. Acc. Chem. Res..

[36] Gu B., Ji W., Patil P.S., Dharmaprakash S.M., Wang H.-T. (2008). Two-photon-induced excited-state absorption: Theory and experiment. Appl. Phys. Lett..

[37] Xu C., Webb W.W. (1996). Measurement of two-photon excitation cross sections of molecular fluorophores with data from 690 to 1050 nm. J. Opt. Soc. Am. B.

[38] Dubinina G.G., Price R.S., Abboud K.A., Wicks G., Wnuk P., Stepanenko Y., Drobizhev M., Rebane A., Schanze K.S. (2012). Phenylene Vinylene Platinum(II) Acetylides with Prodigious Two-Photon Absorption. J. Am. Chem. Soc..

[39] Jiang X.-F., Polavarapu L., Neo S.T., Venkatesan T., Xu Q.-H. (2012). Graphene Oxides as Tunable Broadband Nonlinear Optical Materials for Femtosecond Laser Pulses. J. Phys. Chem. Lett..

[40] Li X., Wang Y., Wang Y., Wang H., Qi X., He J., Xiao S. (2020). Donor-Acceptor Type Reduced Graphene-Oxide and a Tin-Selenide Nanohybrid With Broad and Ultrafast Optical Limiting Properties. Front. Phys..

[41] Husain A., Ganesan A., Sebastian M., Makhseed S. (2021). Large ultrafast nonlinear optical response and excellent optical limiting behaviour in pyrene-conjugated zinc(II) phthalocyanines at a near-infrared wavelength. Dyes Pigm..

