# Supplementary material for: Synthesis and Ultrafast Broadband Optical Limiting Properties of a Two-Branched Twistacene
Source: Molecules. 2022 Jun 1;27(11):3564. doi: 10.3390/molecules27113564 (PMC9181967; doi:10.3390/molecules27113564)
Supplement: Supplementary file 1 [file molecules-27-03564-s001.zip › molecules-1742081-supplementary.pdf]

molecules

MDPI

Supplementary Materials

Synthesis and Ultrafast Broadband Optical Limiting Properties of a Two-Branched Twistacene

Yanbing Han [1], Jinchong Xiao [2], Xingzhi Wu [3], Yuxiao Wang [1], Xueru Zhang [1] and Yinglin Song [1,*]

[1] Department of Physics, Harbin Institute of Technology, Harbin 150001, China; hanyanbing1990@126.com (Y.H.); wangyx@hit.edu.cn (Y.W.); xrzhang@hit.edu.cn (X.Z.)
[2] Key Laboratory of Chemical Biology of Hebei Province, College of Chemistry and Environmental Science, Hebei University, Baoding 071002, China; jcxiaoicas@163.com
[3] Jiangsu Key Laboratory of Micro and Nano Heat Fluid Flow Technology and Energy Application, School of Physical Science and Technology, Suzhou University of Science and Technology, Suzhou 215009, China; wuxingzhi@usts.edu.cn
* Correspondence: ylsong@hit.edu.cn

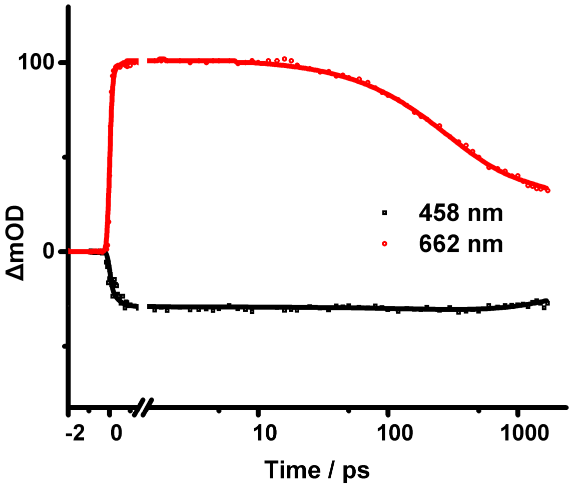

Figure S1. Dynamics traces of **PyDN** at 458 nm and 662 nm. The dots represent the experimental data and solid lines were theoretical fitted curves by global analysis.

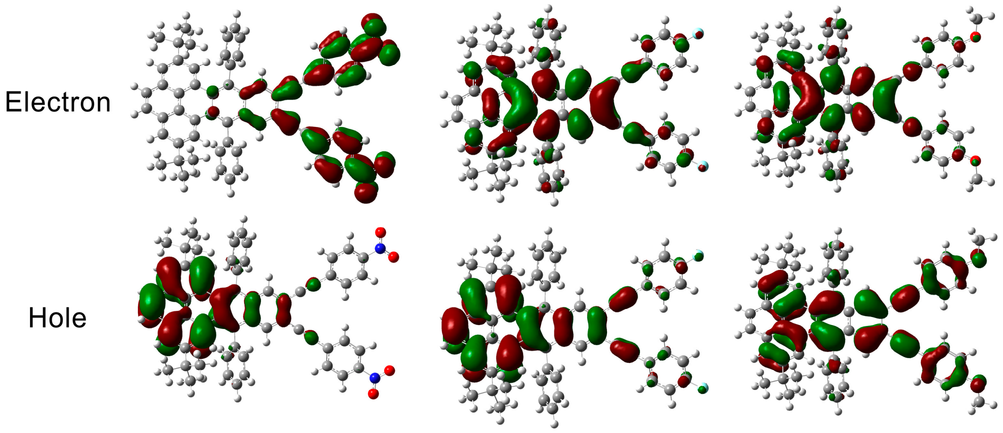

Figure S2. The natural transition orbitals in the first excited state (S1) of designed twistacenes.

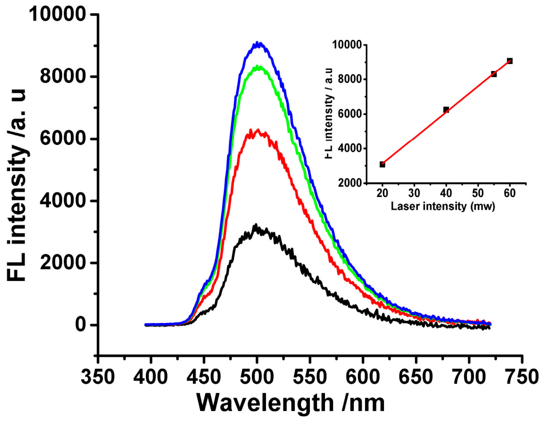

Figure S3. The emission spectra of PyDN in toluene under different incident laser energies at 400 nm. Insets is that linear dependence of one-photon-excited fluorescence intensity on the laser intensity.

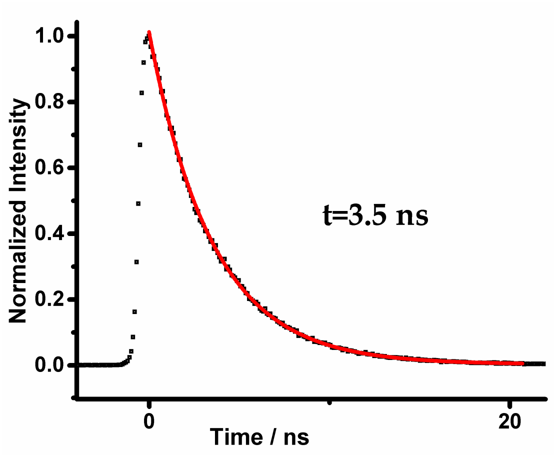

Figure S4. Fluorescence decay of **PyDN** in toluene. The solid line was fitted lifetime of 3.5 ns.

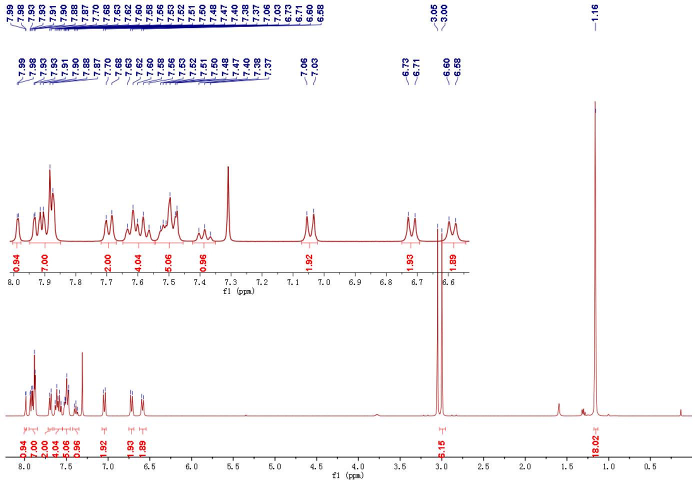

Figure S5. ^{1}H NMR spectrum of **PyDN.**

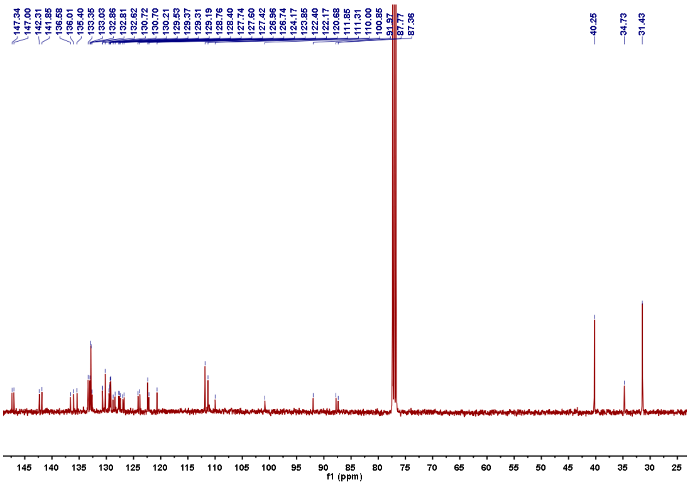

Figure S6. ^{13}C NMR spectrum of **PyDN.**

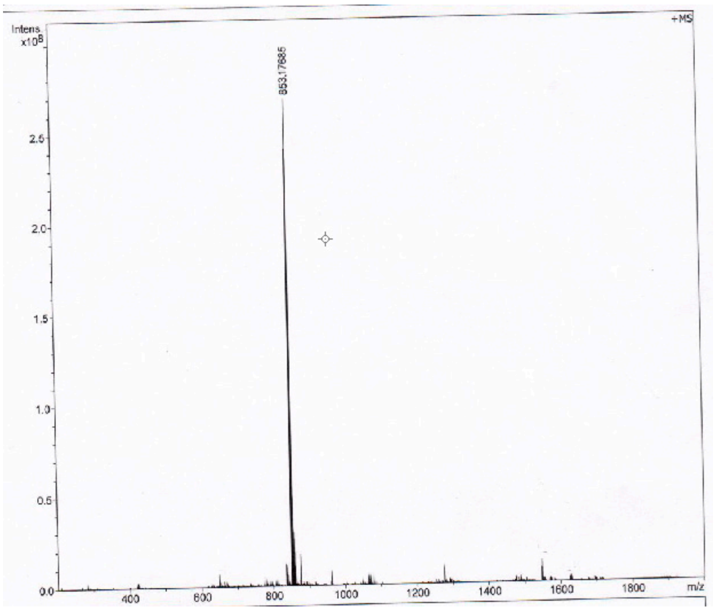

Figure S7. MALDI-TOF spectrum of **PyDN**.
